# Thrombin generation profiling in multiple myeloma: a comprehensive evaluation of prothrombotic state

**DOI:** 10.1097/MBC.0000000000001424

**Published:** 2026-05-03

**Authors:** Ciro Miele, Alessandra Vasco, Luca Manfredi, Immacolata Randa, Roberta Della Pepa, Stella Puzone, Raffaella Addesso, Vittoria Di Vico, Nadia Tinto, Giulia Frisso, Olga Scudiero, Savoia Marcella, Cristina Mazzaccara

**Affiliations:** a Integrated Department of Laboratory and Transfusion Medicine, University of Naples Federico II; b Department of Molecular Medicine and Medical Biotechnologies, University of Naples Federico II; c CEINGE Advanced Biotechnologies Franco Salvatore; d Hematology Unit, Department of Clinical Medicine and Surgery, University of Naples Federico II, Naples; e Casa di Cura S. Michele, Caserta, Italy

**Keywords:** endothelial dysfunction, hypercoagulability, multiple myeloma, venous thromboembolism, thrombin generation

## Abstract

**Objective:**

Venous thromboembolism is a major complication in patients with multiple myeloma (MM), with treatment strategies further increasing thrombotic risk. Conventional coagulation tests (CCT) fail to reflect the in vivo hemostatic balance, whereas thrombin generation assay (TGA) provides a global assessment of thrombin dynamics and may better identify hypercoagulable states in MM.

**Methods:**

To this aim, to evaluated thrombin generation, we enrolled 20 MM patients during follow-up while receiving active MM-treatment: sixteen had immunoglobulin G (IgG) MM, one immunoglobulin A (IgA) MM, one light-chain MM, and two biclonal MM (IgG and IgA). Citrated blood samples were collected under standardized conditions for CCT and TGA. Thrombin generation was measured in platelet poor plasma, both in the absence and presence of thrombomodulin (TM) using the ST Genesia system.

**Results:**

TGA revealed increased peak height (139.75%; r.v. 40–69%), endogenous thrombin potential (107.80%; r.v. 58–78%), and velocity index (153.10%; r.v. 31–62%), along with shortened time to peak (ratio 1.04; r.v. 1.2–1.5). Notably, TM-mediated inhibition of thrombin generation was reduced (16.20%; r.v. 60–76%), indicating protein C pathway dysfunction despite preserved natural anticoagulant levels. This procoagulant state was further supported by elevated fibrinogen and D-dimer, together with increased factor VIII and von Willebrand levels.

**Conclusion:**

Our findings confirm a hypercoagulable state in MM patients, likely related to endothelial injury and systemic inflammation. Impairment of the protein C pathway, as highlighted by TGA, further contributes to this imbalance. In light of the limitations of prothrombin time and activated partial thromboplastin time, TGA provides a more sensitive and informative evaluation of coagulation dynamics in MM patients.

## Introduction

Multiple myeloma (MM) is a hematologic malignancy characterized by the clonal proliferation of plasma cells in the bone marrow with the secretion of a complete monoclonal immunoglobulin or free light chains [1,2]. It accounts for approximately 1% of all cancers and 10–12% of hematologic malignancies [3].

Thrombogenicity in MM is widely acknowledged as a multifactorial process driven by the complex interaction between patient-related risk factors, disease-specific biological mechanisms, and treatment-induced procoagulant effects.

Venous thromboembolism (VTE) is one of the major complications in patients with MM, in addition to cancer-related factors, especially in the first months after diagnosis or at the beginning of Table 1[4]. The new therapeutic agents, including proteasome inhibitors, immunomodulatory drugs (IMIDs) and monoclonal antibodies, although improving clinical outcome of MM, affect the frequency and epidemiology of VTE, reducing survival rate [5]. The rate of VTE depends on the choice of therapy: it is about 2% during monotherapy with IMIDs and can reach 7–26% when IMIDs are associated with corticosteroids or other chemotherapies [6,7]. To prevent immunomodulatory drug-associated thrombosis in MM patients, the International Myeloma Working Group (IMWG), along with the American Society of Clinical Oncology have drawn up guidelines with the aim to assist physicians in the improvement of thromboprophylaxis therapy (aspirin or low molecular weight heparin) in this group of patients [8,9].

**Table 1:** Demographic and clinical characteristics of MM study population

	MM patients (*N* = 20)
Sex, male	10 (50%)
Age at enrollment (years)	61±13
*Male*	62 ± 13
*Female*	59 ± 14
Pro-thrombotic risk factors^*^	17 (85%)
Previous thrombotic events	4 (20%)
Hereditary thrombophilia^Δ^	0 (0%)
Anticoagulant therapy^**^	5 (25%)
Antiplatelet therapy	11 (55%)
MM treatment ^#^	20 (100%)

Data are expressed as: mean ± standard deviation for the continuous variable (age); absolute number and percentage (in parenthesis) for the categorical variables.

MM, multiple myeloma.

*Including one or more of the following factors: obesity, hypertension, hereditary thrombophilia, diabetes mellitus, previous surgical interventions, dyslipidemia, smoking, immobilization, chronic cardiomyopathy.

^**Δ**^ inherited thrombophilia screening included Factor V Leiden and Prothrombin G20210A mutations.

^**^Including heparin or direct oral anticoagulant (DOAC) or coumadin.

^#^Including one or more of the following drugs: immunomodulators, glucocorticoids, erythropoiesis stimulants.

Although the pathophysiology of VTE in MM remains incompletely understood, several factors are known to contribute to the hyperviscosity and prothrombotic state, including elevated levels of circulating paraproteins and increased procoagulant factors such as factor VIII (FVIII), von Willebrand factor (vWF), and fibrinogen [10]. These alterations are associated with the overproduction of inflammatory cytokines by malignant plasma cells, primarily interleukin-6 (IL-6) and vascular endothelial growth factor (VEGF) [11]. Studies have also reported the presence of procoagulant autoantibodies, such as the lupus anticoagulant or anti-protein S antibodies in addition to the acquired activated protein C resistance [12–15]. These findings suggest the need to identify novel and useful biomarkers that accurately reflect the hypercoagulant state in MM patients and assist physicians in determining the optimal duration of VTE prophylaxis, thereby facilitating the development of appropriate algorithms for managing this complication.

Conventional coagulation tests (CCT), such as prothrombin time (PT) and activated partial thromboplastin time (aPTT), are clotting-time–based assays designed primarily to detect fibrin clot formation.

However, these tests do not fully represent the intricate mechanisms governing hemostasis *in vivo,* resulting prolonged when investigating plasma deficient in procoagulant factors, but within the normal range in patients suffering from congenital or acquired deficiencies of physiological anticoagulant factors (protein C, protein S and antithrombin), highlighting the gap existing between *in vivo* and *ex-vivo* coagulation.

To address this shortcoming and fill this gap, in recent years have been developed new generation coagulation tests investigating the dynamism of clot formation, such as thrombin generation assay (TGA) [16]. The latter is a global functional assay measuring the overall thrombin production and its inhibition over time after the addition of tissue factor (TF), phospholipids and calcium. Studies have suggested that thrombin generation (TG) may be a predictive marker of hypercoagulable state in patients with MM and could, thus, identify individuals at risk of thrombosis [17].

In this context, the study provides an observational and methodological description of thrombin generation profiles in MM patients, focusing on alterations in coagulation potential without inferring causal relationships with treatment exposure or individual thrombotic risk.

## Materials and methods

### Study population

A retrospective study was conducted from January to December 2022, including twenty MM patients undergoing MM treatment, according to 2014 International Myeloma Working Group criteria [18], belonging to the Hematology Unit of the Federico II University Hospital of Naples.

At diagnosis, patients underwent needle aspiration or bone marrow biopsy to investigate, by flow cytometry, the presence of abnormal plasma cells. Immunoglobulin G (IgG) MM was diagnosed in 16 patients (80%), immunoglobulin A (IgA) MM in 1 patient (5%), light chain MM in 1 patient (5%), biclonal MM (IgG and IgA) in 2 patients (10%).

Patients were enrolled at follow-up during MM treatment. Detailed information regarding the exact phase of MM treatment for each patient was not available. The following data were recorded for each patient: pro-thrombotic risk factors (including obesity, hypertension, hereditary thrombophilia), previous thrombotic events, diabetes mellitus, previous surgical interventions, dyslipidemia, smoking, immobilization, chronic cardiomyopathy (Table 1). No patient was undergoing chemotherapy.

### Study protocol

Routine biochemical investigations, complete blood count, routine and specialized coagulation tests were carried out. Serum protein electrophoresis (SPE), immunotyping of monoclonal proteins, urine Bence Jones protein (BJP) detection and measurement of serum free light chains (sFLC) were evaluated. Furthermore, global assessment of coagulation was investigated using TGA. Evidence of end-organ damage, as defined by the CRAB criteria [19], was investigated by serum calcium measurement, renal function markers (creatinine, urea, Glomerular Filtration Rate (e-GFR), hemoglobin concentration such as the presence of osteolytic lesions revealed by magnetic resonance imaging (MRI) and/or positron emission tomography–computed tomography (PET/CT). Informed consent was obtained from all participants according to the Helsinki Declaration [20].

### Blood sample

Five fasted blood samples [one with EDTA-K3, 5.4 mg, for hematological investigations, three with trisodium citrate solution (0.109 M, 3.2%) for coagulation tests and one without anticoagulant for serum biochemical investigations] were collected from each patient. Serum for biochemistry investigations, was separated by centrifugation at 3000×*g*/min for 10 min and analyzed by Cobas C 503 (Roche Diagnostics International Ltd, Rotkreuz, Switzerland). Serum protein electrophoresis was performed using Capillary Zone Electrophoresis (CZE) on the Capillarys 2 Flex-Piercing system (Sebia, France). This method allows the identification and quantification of a possible monoclonal protein (MP).

In cases where the serum MP is detected or suspected, immunotyping is conducted using immunosubtraction (ISE) and/or immunofixation (IFE). IFE of serum and urine remains the gold standard for the characterization of MP and BJP. Quantification of serum free light chains (sFLC) was carried out using the N-Latex FLC assay on the Siemens BN ProSpec analyzer via a nephelometric method, enabling the measurement of the free *k* and *λ* light chains secreted by plasma cells. An abnormal *k*/*λ* ratio indicates an excess of one light chain type, serving as an indicator of clonal plasma cell proliferation.

Plasma samples for coagulation assays were obtained after centrifugation at 3000×*g*/min for 10 min and stored at −80∘C until analysis. To assess the hemostatic balance of patients, first and second level coagulation assays, which aPTT, PT, Clauss Fibrinogen, D-dimer, Antithrombin, Protein C, Protein S, Activated Protein C Resistance, Lupus Anticoagulant, Homocysteine and Factor VIII were performed using ACLTOP 550 CTS coagulometer system (Instrumentation Laboratory Company, Bedford, MA, USA). Anti-cardiolipin antibodies (aCL-IgG/IgM), anti-β2-glycoprotein I antibodies (aβ2GPI-IgG/IgM) and von Willebrand antigen (vW:Ag) tests, were conducted by chemiluminescence immunoassay using HemosIL AcuStar (Instrumentation Laboratory Company, Bedford, MA, USA). Blood count was performed on peripheral blood by routine procedures on Sysmex XN-3100 Automated Hematology Analyzer (Sysmex America, Inc). All the procedures took place according to the manufacturer's recommendation.

### Thrombin generation assay

Comprehensive assessment of coagulation was performed on thawed platelet-poor plasma (PPP) using TGA carried out on the fully automated ST Genesia system (STG, Stago, Asnières-sur-Seine, France). Thrombin generation was evaluated with the STG-ThromboScreen (STG-TS) reagent kit, containing a mixture of phospholipids and a medium picomolar concentration of human TF, in the presence and absence of thrombomodulin (TM). The parameters recorded, included: lag time, representing the time needed to thrombin formation after coagulation initiation; peak height, which is the maximum thrombin generated during the assay; time to peak, the time it takes for thrombin to reach its maximum concentration; velocity index, a measure of the rate of thrombin generation; endogenous thrombin potential (ETP), defined as the area under the thrombogram curve and showing the total amount of thrombin produced; ETP inhibition, representing the degree of ETP suppression mediated by TM [21] and automatically calculated by the instrument software according to the formula: (ETP without TM – ETP with TM)/(ETP without TM). All tested plasma specimen was processed concurrently with a normal reference plasma (STG-Ref Plasma), and the instrument software automatically provided patient values normalized to this control [22]. All analyzes were carried out in full accordance with the manufacturer's instructions.

## Results

Demographic characteristics, thrombotic risk evaluation, MM treatment of patients studied are showed in Table 1. At recruitment, four patients (20%) reported history of previous thrombotic events, data in agreement with literature [23], and 17 patients (85%) showed pro-thrombotic risk factors. Eleven patients (55%) received antiplatelet drugs and 5 patients (25%) underwent anticoagulant therapy. MM treatment was administered in all patients, including ten patients undergoing therapy with immunomodulators, glucocorticoids and erythropoiesis stimulants; five patients undergoing therapy both with immunomodulators and glucocorticoids; four patients underwent only glucocorticoids; one patient undergoing therapy both with glucocorticoids and erythropoiesis stimulants.

The characterization of MM population including MC typing and quantification, the concentration of sFLC, BJP presence, when detectable, and the disease stage, based on both Durie and Salmon score according to the International Staging System are reported in Table 2[24].

**Table 2:** Characterization of the MM study population

MC (g/l)	*k* FLC (r.v. 6.7–22.4 mg/l)	*λ* FLC (r.v. 8.3–27.0 mg/l)	*k*/ *λ* (r.v. 0.31–1.56)	BJP	Disease stage (ISS)^a^
*k* (1.6)	7200.0↑	4.5↓	1600.0↑	*k*	III
IgG *λ* (1.0)	15.6	59.7↑	0.26↓	n.p.	I
IgG *k* (17.0)	90.4↑	6.2↓	14.6↑	n.p.	III
IgG *λ* (28.0)	16.4	22.9	0.72	n.p.	II
IgA *k* (3.8) and IgG *k* (1.2)	41.4↑	36.6↑	1.13	*k*	II
IgG *λ* (57.0)	3.3↓	261↑	0.01↓	λ	I
IgG *λ* (45) and *λ* (1.0)	7.5	38.3↑	0.19↓	n.p.	II
IgG *k* (1.3)	3.8↓	3.8↓	1.00	n.p.	III
IgG *k* (1.0)	3.7↓	4.4↓	0.84	n.p.	III
IgG *k* (3.7)	14.2	15.4	0.92	n.a.	III
IgG *k* (45.0)	193↑	17.6	11.0↑	*k*	II
IgG *k* (96.0)	4.5	2.4↓	1.87↑	n.a.	III
IgA *k* (19) and IgG *k* (2.0)	403↑	1.5↓	269↑	*k*	II
IgG *λ* (32.0)	4.7↓	37.2↑	0.13↓	*λ*	III
IgA *λ* (63.0)	2.7↓	158↑	0.02↓	*λ*	III
IgG *k* (1.6)	7.0	7.9↓	0.89	n.p.	I
IgG *k* (77)	32.7↑	12.9	2.53↑	*k*	II
IgG *k* (13)	7.1	6.2↓	1.14	n.a.	II
IgG *λ* (4.1) and IgG *k* (9.8)	23.5↑	26.3	0.89	*k*	II
IgG k (8.6)	205↑	7.6↓	27.0↑	n.p.	II

MM, multiple myeloma; MC, monoclonal component; FLC, free light chain; BJP, Bence Jones protein; n.p., not present; n.a., not analyzed.

a based on both Durie and Salmon score and ISS (International Staging System) [23]. Arrows indicate values outside the reference range.

Results for routine biochemical investigations and complete blood count are reported in Table 3 and 4, respectively. Routine and extended coagulation tests were also investigated to evaluate the hemostatic balance (Table 5). Kolmogorov–Smirnov test was performed to test for normality of distribution. Median, 25h and 75h percentile (interquartile range) were reported for non-parametric distribution and mean ± standard deviation for normal distribution.

**Table 3:** Biochemical parameters in the MM study population

Parameter	MM patients (*N* = 20)
Sodium (Na^+^), mmol/l (r.v. 136–145)	140 (137–142)
Potassium (K^+^), mmol/l (r.v. 3.5–5.1)	4.2 (3.9–4.5)
Calcium (Ca^2+^), mg/dl (r.v. 8.4–10.2)	9.3 (8.6–9.6)
Creatinine, mg/dl (r.v. 0.72–1.25)	0.8 (0.7–1.0)
e-GFR, ml/min/1.73 mq (r.v. >90)	86.0 (70.7–98.2)
Urea, mg/dl (r.v. 18–55)	43.5 (31.2–56.7)
Total Protein, g/dl (r.v. 6.4–8.3)	7.3 (6.5–9.1)
Albumin, g/dl (r.v. 3.5–5.2)	3.9 (3.7–4.3)

Data are expressed as median and 25 h to 75 h percentile (interquartile range).

e-GFR, estimated glomerular filtration rate, using CKD-EPI equation.

**Table 4:** Hematological parameters in the MM study population

	MM male patients (*N* = 10)	MM female patients (*N* = 10)
WBC (×10^3^/μl) (r.v. 4.5–11.0)	6.54 ± 3.10	7.29 ± 2.08
RBC (x10^6^/μl) (r.v. F: 4.0–5.0; M: 4.5–5.6)	3.97 ± 0.55	4.06 ± 0.70
Hemoglobin (g/dl) (r.v. F: 12.0–15.5; M: 13.0–16.5)	12.50 ± 1.75	11.84 ± 2.53
HCT (%) (r.v. F: 35.0–48.0; M: 37.0–50.0)	37.70 ± 5.10	36.63 ± 6.48
PLT (×10^3^/μl) (r.v. 150–450)	190.70 ± 67.5	217.50 ± 36.70

Data are expressed as mean ± standard deviation.

WBC, white blood cell; RBC, red blood cell; HCT, hematocrit; PLT, platelet count.

**Table 5:** Routine and extended coagulation tests in the MM study population

Parameters	Results
PT (ratio) (r.v. 0.8–1.20)	0.99 (0.93–1.07)
aPTT (ratio) (r.v. 0.8–1.20)	1.04 (0.98–1.08)
Fibrinogen (mg/dl) (r.v. 160–350)	**414.00** (327.00–468.00)
AT (%) (r.v. 70–120)	112.50 (99.50–123.50)
Protein C (%) (r.v. >62)	119.50 (91.25–147.00)
Protein S (%) (r.v. >58)	80.50 (70.75–98.50)
D-Dimer (ng/ml) (r.v. 0–500)	**536.00**(444.50–969.50)
FVIII:C (%) (r.v. 50–130)	**149.00**(120.25–182.50)
VWF:Ag (%) (r.v. blood type 0: 44–116 non-0 blood type: 63–159)	**162.05** (116.15–199.50)
APC resistance (NTR) (r.v. >0.75)	1.08 (0.99–1.11)
Homocysteine (μmol/l) (r.v. 5–15)	8.35 (6.83–15.72)
Lupus anticoagulant (ratio) (r.v. 0.8–1.20)	1.02 (0.94–1.10)
Anti-Cardiolipin IgG (U/ml) (r.v. <20)	0.00 (0.00–1.25)
Anti-cardiolipin IgM (U/ml) (r.v. <20)	0.70 (0.30–1.35)
Anti-β2-glycoprotein I IgG (U/ml) (r.v. < 20)	2.40 (1.82–2.87)
Anti-β2-glycoprotein I IgM (U/ml) (r.v. < 20)	0.15 (0.00–2–0.60)

Data are expressed as mean ± standard deviation. Bold values represent values above the r.v.

PT, prothrombin time; aPTT, activated partial thromboplastin time; APC, activated protein C; AT, antithrombin; FVIII:C, coagulative factor VIII; VWF:Ag, von Willebrand factor antigen.

As shown in Table 5, elevated levels of fibrinogen and D-dimer suggest ongoing activation of the coagulation system. This is further supported by increased plasma concentrations of FVIII and vWF antigen. As reported in literature, these factors contribute to a heightened tendency for clot formation increasing the risk of thrombotic events such as deep vein thrombosis or pulmonary embolism [25,26].

Testing for deficiencies of the natural anticoagulants (Protein C, Protein S and Antithrombin) and the assessment of Resistance to Activated Protein C (APCR) did not show any alteration involving the physiological coagulation inhibition pathway in these patients. Furthermore, the results for screening of anti-phospholipid antibody syndrome (APS) and homocysteine (HCY) levels were also negative.

Collectively, these findings exclude the presence of other thrombogenic conditions beyond MM that could confound the interpretation of our results

**Fig. 1:**
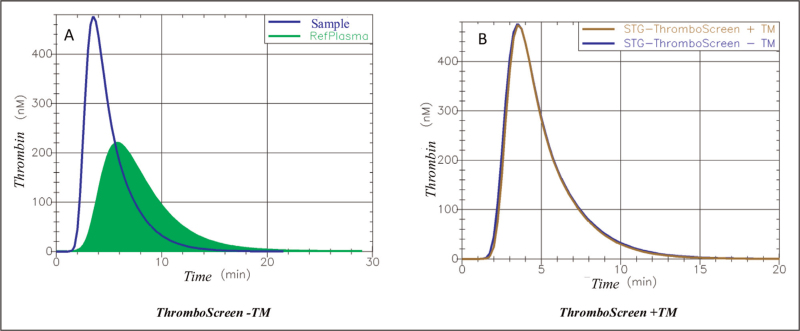
Representative thrombograms with and without TM, obtained using ST Genesia analyzer in a MM patient. (a) Thrombogram curve generated in the absence of TM. (b) Thrombogram curve generated when exogenous TM is added to patients’ plasma and showing no inhibition effect of TM. TM, thrombomodulin; TGA, thrombin generation assay.

Thrombin generation parameters without TM addition, highlighted an increase of peak height, endogenous thrombin potential (ETP) and velocity index, along with a shortened time to peak, indicating an enhanced and accelerated thrombin generation. The addition of TM, a key modulator of physiological anticoagulant activity, revealed a reduced inhibition of TG as pointed out by ETP Inhibition parameter (Table 6, Fig. 1). Table 6 shows Thrombin Generation parameters and their reference value without and with TM in MM study population [27]. These data suggest a reduced functional capacity of the protein C pathway, highlighting an impairment in the physiological regulation of coagulation in MM patients, despite the normal values of Protein C, Protein S and Antithrombin.

**Table 6:** Thrombin Generation parameters without and with TM in MM study population

Parameter	MM patients (*N* = 20)
Lag time (ratio) (r.v. 1.1–1.4)	1.11 (0.99–1.23)
Peak height (%) (r.v. 40–69)	**139.75**(125.00–154.20)
Time to peak (ratio) (r.v. 1.2–1.5)	**1.04** (0.97–1.07)
Velocity index (%) (r.v. 31–62)	**153.10** (130.32–221.80)
ETP (%) (r.v. 58–78)	**107.80** (90.76–120.17)
ETP inhibition (%) (r.v. 60–76)	**16.20** (10.35–24.33)

Data are expressed as median and 25 h to 75 h percentile (interquartile range). Reference values (r.v.) were adapted from Calzavarini *et al.*[27], reporting the minimum and maximum values observed across both sexes in healthy subjects ≥50 years. ETP Inhibition, is obtained when TM is added to patients’ plasma. Bold values represent values above the r.v.

ETP, endogenous thrombin potential; TM, thrombomodulin.

## Discussion and conclusion

The mechanisms underlying VTE in cancer are heterogeneous and influenced by a multitude of factors, including tissue factor overexpression on circulating cancer cells, the release of procoagulant microparticles from malignant cells, enhanced interactions between tumor cells and platelets. Additionally, the formation of DNA-based neutrophil extracellular traps (NETs) and the prothrombotic effects of certain cancer therapies further contribute to this hypercoagulable state [28]. These processes highlight the complex interplay between malignancy and thrombosis, presenting significant challenges in the management of cancer-associated VTE. The prothrombotic pathway, observed in our patient group, appears to reflect a host response to the tumor, characterized by inflammation, tissue necrosis, and hemodynamic alterations. These processes collectively impact all three components of Virchow's triad: hypercoagulability, vessel wall injury/endothelial damage, and stasis [29,30].

In addition, tumor-specific clot-promoting mechanisms, such as expression of procoagulant and fibrinolytic activities by the tumor cells and interaction with endothelial cells and blood cells, play a role in the pathogenesis. Multiple risk factors for thrombosis have been identified in patients with MM, including treatment with thalidomide or lenalidomide in combination with dexamethasone, as well as multi-agent chemotherapy. These therapeutic approaches further exacerbate the hypercoagulable state, which represents a significant factor underlying morbidity and mortality in this patient population. In order to mitigate thrombotic events and support clinicians in identifying patients who may benefit from extended antithrombotic prophylaxis, several risk stratification models have been developed, including IMWG, IMPEDE-VTE, PRISM and SAVED [31]. Nevertheless, to date, it is still difficult to detect a reliable biomarker capable of adequately reflecting a prothrombotic state in patients with MM and enhancing risk stratification models. [32]. Therefore, to investigate the hypercoagulable state in these patients, thrombin generation was assessed using platelet-poor plasma from 20 MM patients, employing the ST Genesia system, a novel fully automated TGA. Our data confirm previously findings by Velasco-Rodriguez *et al.*[33], suggesting hypercoagulable state in untreated MM patients, as indicated by thrombin generation profiles, showing impairment of the protein C anticoagulant pathway. Specifically, TM-mediated inhibition failed to significantly reduce endogenous thrombin potential in MM patients, despite the absence of congenital or acquired deficiencies in the natural anticoagulant system. Elevated thrombin concentrations in MM patients contribute to an imbalance in physiological anticoagulant mechanisms, thereby promoting a prothrombotic state [34]. This is likely related to endothelial injury, a hallmark of the disease, which disrupts critical anticoagulant pathways [35]. This procoagulant state was further supported by elevated levels of fibrinogen and D-dimer, alongside increased FVIII and vWF antigen, which serve as indicators of systemic inflammation and endothelial dysfunction, as already pointed out in literature [36]. Thrombomodulin plays a crucial role as a natural anticoagulant and in preserving an anti-inflammatory endothelial environment [37]. However, TM expression is downregulated in vascular injury-related disorders, further exacerbating both the procoagulant and inflammatory state [38]. Giri *et al.* investigated the anti-inflammatory role of TM, demonstrating that TM-deficient endothelial cells exhibit increased thrombin activity and heightened responsiveness to proinflammatory stimuli, resulting in elevated secretion of vWF [39].

These findings highlight TM as a key regulator of procoagulant and proinflammatory molecule expression [39]. Since hematological diseases are known to promote a prothrombotic state through endothelial injury, the impaired PC system, observed in our MM patient cohort could be attributed to the disruption of TM expression [40].

Previous studies have reported increased TM release from endothelial cells into the bloodstream in MM patients. This occurs due to TM shedding from the surface of injured endothelial cells and the downregulation of TM expression driven by systemic inflammation, ultimately altering the physiological anticoagulant properties of the endothelium [41]. These alterations contribute to a prothrombotic state that is often not detected by conventional coagulation assays commonly used in clinical practice, such as PT and aPTT, which are primarily sensitive to procoagulant factor deficiencies rather than hypercoagulability [42]. Conversely, the TGA provides a more comprehensive assessment of the hemostatic balance in MM patients and may help identify those at increased risk of thrombosis [43]. Incorporating advanced hemostasis laboratory techniques into clinical practice may improve the ability to recognize high-risk patients and support more effective prevention strategies of thromboembolic events within this population.

## Limitation

The main limitations of this study include the small sample size. Future studies with larger patient cohorts are needed to validate the clinical utility of thrombin generation assay in MM patients and to define its role in prothrombotic risk evaluation.

## Acknowledgements

None.

No funding was received for the present study.

Author contributions: Conceptualization was carried out by C.M. (Ciro Miele), C.M. (Cristina Mazzaccara), and M.S. Investigation was conducted by C.M. (Ciro Miele), A.V., L.M., I.R., and S.P. Data curation was undertaken by M.S., C.M. (Cristina Mazzaccara), C.M. (Ciro Miele), G.F., N.T., O.S., R.D.P., V.D.V and R.A. The original draft was written by C.M. (Ciro Miele), C.M. (Cristina Mazzaccara), A.V and M.S. Writing – review and editing were performed by C.M. (Ciro Miele) and C.M. (Cristina Mazzaccara). Supervision was provided by C.M. (Ciro Miele) and C.M. (Cristina Mazzaccara). All authors have read and agreed to the published version of the manuscript.

### Conflicts of interest

There are no conflicts of interest.
